# Primitive Fitting Based on the Efficient multiBaySAC Algorithm

**DOI:** 10.1371/journal.pone.0117341

**Published:** 2015-03-17

**Authors:** Zhizhong Kang, Zhen Li

**Affiliations:** School of Land Science and Technology, China University of Geosciences, No. 29 Xueyuan Road, Haidian District, Beijing, 100083, China; Fondazione Edmund Mach, Research and Innovation Centre, ITALY

## Abstract

Although RANSAC is proven to be robust, the original RANSAC algorithm selects hypothesis sets at random, generating numerous iterations and high computational costs because many hypothesis sets are contaminated with outliers. This paper presents a conditional sampling method, multiBaySAC (Bayes SAmple Consensus), that fuses the BaySAC algorithm with candidate model parameters statistical testing for unorganized 3D point clouds to fit multiple primitives. This paper first presents a statistical testing algorithm for a candidate model parameter histogram to detect potential primitives. As the detected initial primitives were optimized using a parallel strategy rather than a sequential one, every data point in the multiBaySAC algorithm was assigned to multiple prior inlier probabilities for initial multiple primitives. Each prior inlier probability determined the probability that a point belongs to the corresponding primitive. We then implemented in parallel a conditional sampling method: BaySAC. With each iteration of the hypothesis testing process, hypothesis sets with the highest inlier probabilities were selected and verified for the existence of multiple primitives, revealing the fitting for multiple primitives. Moreover, the updated version of the initial probability was implemented based on a memorable form of Bayes’ Theorem, which describes the relationship between prior and posterior probabilities of a data point by determining whether the hypothesis set to which a data point belongs is correct. The proposed approach was tested using real and synthetic point clouds. The results show that the proposed multiBaySAC algorithm can achieve a high computational efficiency (averaging 34% higher than the efficiency of the sequential RANSAC method) and fitting accuracy (exhibiting good performance in the intersection of two primitives), whereas the sequential RANSAC framework clearly suffers from over- and under-segmentation problems. Future work will aim at further optimizing this strategy through its application to other problems such as multiple point cloud co-registration and multiple image matching.

## Introduction

Primitive fitting mechanisms serve as a central component of remote sensing applications, such as 3D modeling and as-built surveys. Primitive fitting is primarily conducted to estimate model parameters from raw data that are contaminated by outliers. Hence, the majority of existing techniques focus on the robust estimation of primitive parameters (e.g., 3D Hough Transforms [1], Region Growing [2]-[3], Expectation Maximization algorithms [4–6], Generalized PCA algorithms [7] or Ensembles [8]).

A well-regarded technique for the segmentation and robust model fitting of range data is the statistical framework of RANdom SAmple Consensus (RANSAC) [9]. Rather than using as much data possible to obtain an initial solution and subsequently attempting to eliminate invalid data points, the RANSAC method tests several minimal random subsets and evaluates each subset’s model fit. Because it is proven to be capable of addressing more than 50% of all outliers, RANSAC is increasingly being utilized for primitives fitting image matching, and for other improved algorithms. Torr and Zisserman [10] applied the robust estimation method of Maximum Likelihood Estimation SAmple Consensus (MLESAC) to identify best-fitting roof models in a model-driven manner, and Torr and Davidson [11] presented IMPortance sampling and random SAmple Consensus (IMPSAC) methods, which use a hierarchical resampling algorithm. R-RANSAC [12] involves increasing the model parameter estimation speed through hypothesis evaluation randomization processes resulting from numerous erroneous model parameters that arise from contaminated samples evaluated in RANSAC. Schnabel et al. [13] improved the efficiency of RANSAC through local point selection and through the incorporation of a simplified score function. An optimized and randomized version of RANSAC [14] based on the estimation of two probabilities that characterize the problem and critically affect the design of the optimal strategy was also proposed. Cheng and Lai [15] presented a consensus sampling technique for fast and robust model fitting by improving consensus sampling, model evaluation and robust standard deviation estimation approaches. In addition, Gallo et al. [16] presented a robust estimation method called CC-RANSAC that modifies the RANSAC algorithm by only considering the largest connected components of inliers in evaluating the fitness of a candidate plane. A spatio-temporal RANSAC method that uses 3D video data was also developed for the purposes of planar surface estimation [17].

However, the original RANSAC algorithm assumes constant prior probabilities for data points and selects initial datasets randomly, which likely generates additional iterations and high computation costs when the hypothesis set is contaminated by several outliers. Moreover, the original RANSAC method is limited by the assumption that a single model accounts for all data inliers. The sequential strategy (sequential RANSAC) has been widely used to manage multiple models (or different instances of the same model) [18]. Bartoli [19] presented a multiple hypothesis version of RANSAC that modifies the original algorithm by maximizing the plane fitting probability and that allows for the piecewise segmentation of overlapping planes. Papalazarou et al. [20] also presented a complex curve estimation method based on Sparse-Plus-Dense RANSAC that identifies multiple model instances in two- and three-dimensional data. However, inaccurate inlier detection during initial (or subsequent) parameter estimation substantially increases the parameter estimate instability of remaining models.

To address these shortcomings of the original RANSAC method, this paper presents the multiBaySAC algorithm of multi-primitive fitting that applies a 3D point cloud based on the BaySAC algorithm (Bayes Sample Consensus) [21]. The proposed algorithm implements a conditional sampling strategy that always selects the minimum number of data points required with the highest inlier probabilities as a hypothesis set thereby reducing the number of iterations needed to identify a good model. A novel statistical testing method for candidate model parameters was first presented to detect initial multiple primitives. The detected primitives were then optimized using a parallel rather than sequential strategy so that every data point was assigned to multiple prior probabilities for initial multiple primitives. with a conditional sampling method, BaySAC, implemented in parallel. With each iteration of the hypothesis testing process, the hypothesis sets with the highest prior probabilities were selected and verified for the presence of multiple primitives, thus determining the fit of multiple primitives. Moreover, the updated probability was applied using the simplified Bayes’ formula.

The main contributions of this paper are listed in Section II, which introduces the multiBaySAC method. Section III discusses our test results, and in Section IV, we offer our conclusions and suggestions for further research.

### MultiBaySAC

As a well-regarded technique for the segmentation and robust model fitting of range data, RANSAC is proven to be capable of addressing more than 50% of all outliers.

### A) RANSAC framework

RANSAC is an iterative method for estimating mathematical model parameters from observed data that contain outliers. RANSAC assumes that when an usually small set of inliers is involved, a procedure that estimates model parameters that optimally explain or fit these data can be applied.

The RANSAC paradigm is governed by three parameters: (1) the error tolerance used to determine whether a point is compatible with a model; (2) the number of subsets to test; and (3) the number, t, of compatible points needed for a model to be deemed correct. Definitions for these three parameters are provided in the original RANSAC paper [9].

The RANSAC algorithm input used in this study is a series of laser points, thus fitting the model to the primitives and to confidence parameters. RANSAC fits the primitive model in the following manner:

1) Select a random subset of laser points as the hypothetical inliers;
2) A primitive model is fitted to the set of hypothetical inliers;
3) All other data are then tested against the fitted model. Those points that fit the estimated model well, according to a model-specific loss function (e.g. the distance between laser point i and the fitted primitive), are considered as part of the consensus set;
4) Select the primitive model that is consistent with most data points as the best model;
5) Repeat the hypothesis testing process until the sampling iteration number reaches the predefined threshold T;
6) Use all inlier points to compute the optimal primitive model using the least-squares adjustment.

The computational cost of RANSAC is proportional to the number of iterations involved, which corresponds to the number of hypothesis sets that are chosen before a good enough model is found. The original RANSAC hypothesis set sampling strategy assumes that all data points are equally probable. Therefore, the order in which they are considered is irrelevant, producing numerous iterations and high computational costs as many hypothesis sets are contaminated with outliers. Moreover, the original RANSAC method is limited by the assumption that a single model accounts for all data inliers.

To tackle these issues of the original RANSAC method, we present the multiBaySAC algorithm of multi-primitive fitting from a 3D point cloud based on the BaySAC algorithm, which implements parallel rather than sequential multiple model detection.

### B) Algorithm overview

**Fig 1:**
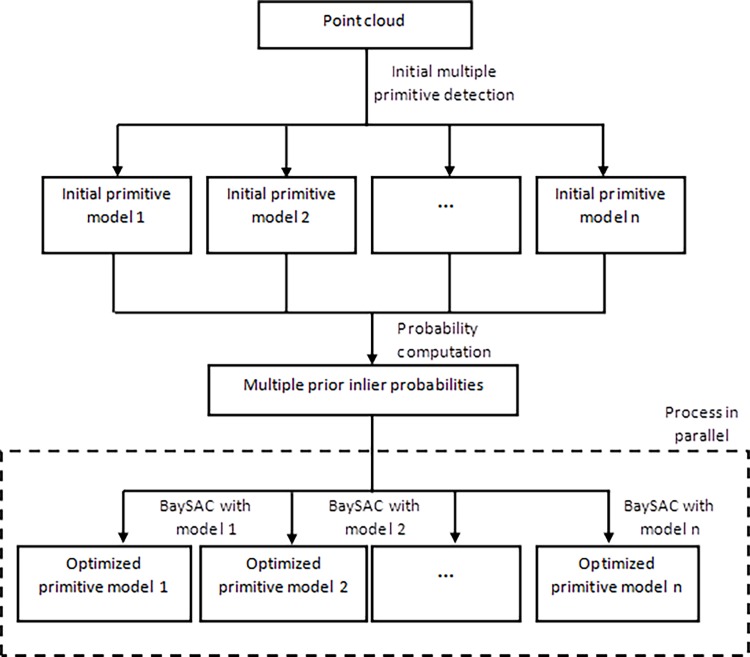
multiBaySAC flowchart. A statistical testing algorithm for the candidate parameter set is performed to detect initial multiple primitives from the scattered point cloud so that every data point in the point cloud is assigned multiple prior inlier probabilities for the detected initial multiple primitives. With multiple prior inlier probabilities the fitting of multiple models based on the BaySAC process is then conducted simultaneously to estimate the optimized primitive models.

Fig. 1 presents the multiBaySAC flowchart. First, a statistical testing algorithm for the candidate parameter set was applied to detect initial multiple primitives in the scattered point cloud. Every data point in the point cloud was then assigned to multiple prior inlier probabilities for detected initial multiple primitives. Each lists the probability that a point belongs to the corresponding primitive. Using multiple prior inlier probabilities, we then applied the fitting of multiple models using the BaySAC process in parallel to estimate the optimized primitive models.

**Fig 2:**
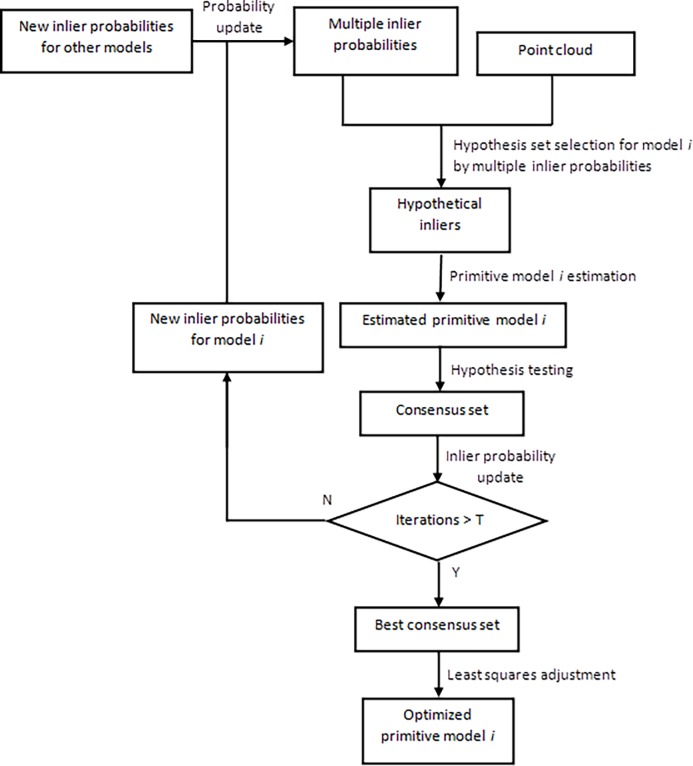
BaySAC process flowchart for the fitting of primitive model *i*. Use n data points with the highest inlier probabilities as the hypothesis set to fit primitive model *i*; evaluate all data points w.r.t. primitive model *i* and update the inlier probabilities of each data point concerning primitive model *i* in the hypothesis set via Bayes’ rule; update the multiple inlier probabilities using the new inlier probabilities for model *i* as well as the new inlier probabilities for other models updated through other BaySAC processes running in parallel; repeat the hypothesis testing process until the sampling number reaches the defined threshold; using all inlier points, compute the optimal model parameters through least-squares adjustment.

The BaySAC procedure for the fitting of primitive model *i* is illustrated in Fig. 2. The algorithm proceeds as follows:

1) Select n data points with the highest inlier probabilities as the hypothesis set;
The data point with an inlier probability for hypothesis primitive *i* is considered the highest not only among all inlier probabilities concerning the current primitive, but also among the multiple inlier probabilities assigned to the considered point.
2) Fix primitive model *i* corresponding to the n chosen data points;
3) Evaluate all of the data points of w.r.t. primitive model *i* and determine inliers and outliers based on a preset threshold;
4) Update the inlier probabilities of each data point concerned with primitive model *i* in the hypothesis set using Bayes’ rule;
5) Update the multiple inlier probabilities using new inlier probabilities for model *i* and new inlier probabilities for other models that were updated through other simultaneous BaySAC processes.
6) Repeat steps 1 to 5 until the sampling number reaches T:
  $$\text{T}=\frac{\left(\log\left(1-p\right)\right)}{\log\left(1-{\left(1-\varepsilon \right)}^{n}\right)}$$ (1)
  Where p is the confidence probability and *ε* is the outlier rate that is computed as the number of outliers in the dataset divided by the number of data points.
7) Select the primitive model that is consistent with the most data points as the best model *i*;
8) Using all inlier points, compute the optimal model parameters via least-squares adjustment.

### C) Primitive parameterization

**Fig 3:**
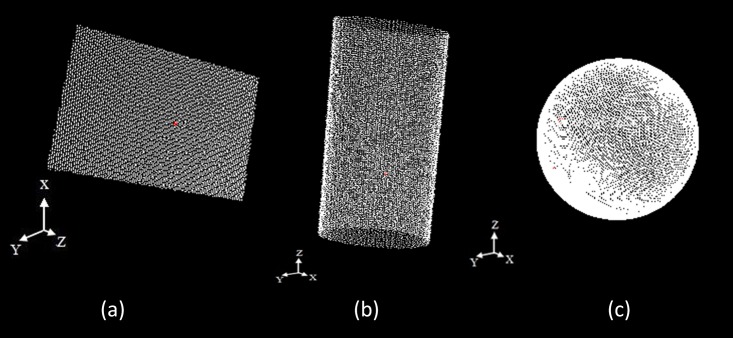
Geometric primitives of interest. Three common geometric primitives are considerated: **(a)** plane, **(b)** cylinder, (**c)** sphere.

In this paper, three common geometric primitives, i.e., planes, cylinders and spheres (Fig. 3), are considered. We describe the primitive parameterization as follows.

**Fig 4:**
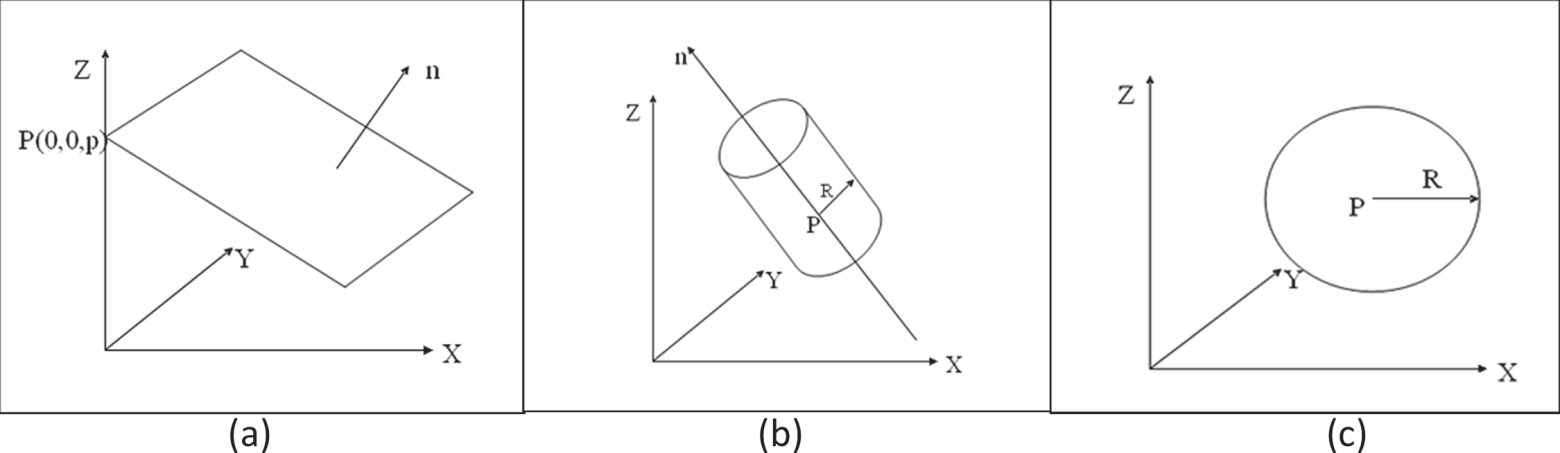
Primitive parameterization. The primitive parameterization is described as follows **(a)** A singularity free representation (Hesse form) of a plane: the planar primitive is represented by the normal vector and perpendicular distance from the origin **(b)** An infinite cylinder represented by the axis of the cylinder, the point closest to the origin and the radius **(c)** A sphere defined by its center point and radius.

**a) Planes.** A singularity-free representation of a plane [22] that describes a plane using the normal vector $\vec{n}={\left[\begin{array}{ccc} n_{x} & n_{y} & n_{z} \end{array}\right]}^{T}$ and the perpendicular distance from the origin *ρ* (Fig. 4A) was employed. This representation is also known as the Hesse form of the plane. Equation (2) is the full expression for the parameterization.

$$Xn_{x}+Yn_{y}+Zn_{z}-\rho =0$$ (2)

Because there can be only three degrees of freedom in a plane, we impose a constraint on the length of the normal vector $\vec{n}$:

$$\sqrt{{n_{x}}^{2}+{n_{y}}^{2}+{n_{z}}^{2}}=1.$$ (3)

**b) Cylinders.** In many cases, it could be quite difficult to accurately determine the start and end points of cylinders based on point clouds due to the presence of occlusions and point spacing. For this reason, we employed a parameterization for the infinite cylinder (Fig. 4B). We thus, represented a cylinder using seven parameters: three for the cylinder axis, three for the cylinder axis point and one for the radius. The full expression is as follows:

$${\left(X-a\right)}^{2}+{\left(Y-b\right)}^{2}+{\left(Z-c\right)}^{2}-{\left(i\left(X-a\right)+j\left(Y-b\right)+k\left(Z-c\right)\right)}^{2}=R^{2}$$ (4)

where (*a*, *b*, *c*) denotes a point on the cylinder axis, (*i*, *j*, *k*) represents the cylinder axis, and *R* is the cylinder radius.

Because the cylinder includes only five degrees of freedom, we have two constraints:

$$\begin{array}{c} \left\|\vec{l}\right\|=1\\ \vec{l}\cdot C=0\;, \end{array}$$ (5)

where $\vec{l}$ denotes the cylinder axis and *C* denotes the point closest to the origin.

**c) Spheres.** We define a sphere using its center point and its radius (Fig. 4C) as follows:

$${\left(X-a\right)}^{2}+{\left(Y-b\right)}^{2}+{\left(Z-c\right)}^{2}=R^{2}$$ (6)

where (*a*, *b*, *c*) denotes the coordinates of the center point and *R* represents the sphere radius.

### D) Initial multiple primitive detection based on candidate model parameter statistical testing

Because the mathematical models of primitives to be fitted are determinate, their parameters should be convergent when calculated using consecutive inlier sets. Therefore, we presented a statistical testing algorithm for the candidate parameter set to detect initial multiple primitives. The proposed statistical testing process is implemented using a histogram that illustrates the distribution of the discrete hypothesis model parameter sets that are computed during different iterations and using the degree of convergence of each considered candidate parameter set, describing how other sets converge to it. The degree of convergence of a bin in the histogram is calculated as the number of parameter sets in that bin divided by the total number of parameter sets.

To determine the degree of convergence of the three geometric primitives in Fig. 3, a suitable geometrical measure for each primitive is listed below. We construct a vector for each set of parameters and computed the Euclidian distances between different vectors to determine the deviation between the hypothesis parameter sets that are computed during consecutive iterations.

**Fig 5:**
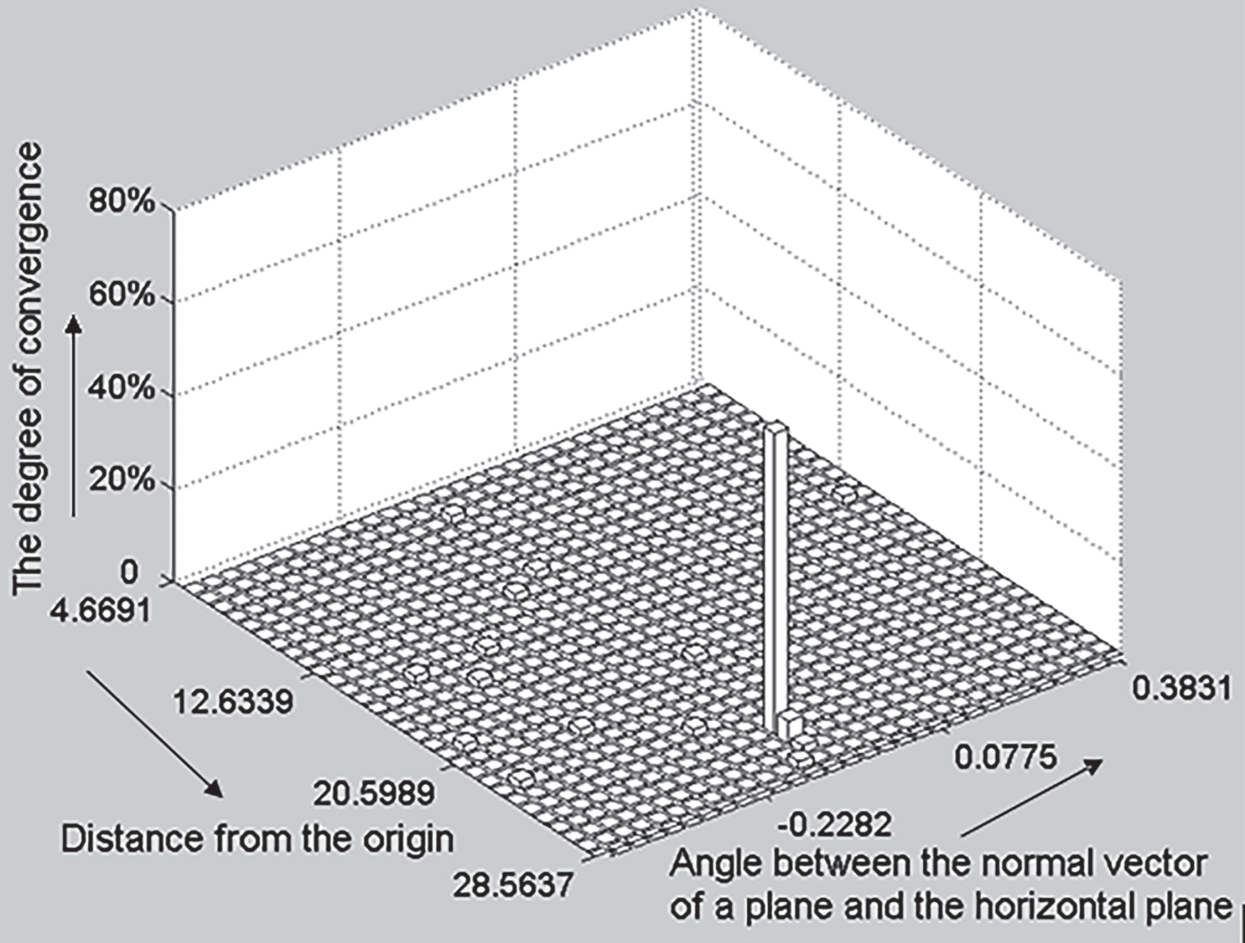
The 2D histogram of a plane. The horizontal and vertical axes denote the angle between the normal vector n of a plane and horizontal plane and the perpendicular distance from the origin respectively; the upright axis represents the convergence degree of each candidate parameter set. This degree of candidate parameter set convergence in the histogram is calculated as the number of parameter sets that converge to the candidate parameter set divided by the total number of parameter sets.

**a) The 2D histogram of a plane.** In this paper, we use the Hesse form of the plane; therefore, differences between hypothesis planar parameters are described in terms of the normal vector $\vec{n}={\left[\begin{array}{ccc} n_{x} & n_{y} & n_{z} \end{array}\right]}^{T}$ and perpendicular distance from origin *ρ*. In Fig. 5, the horizontal and vertical axes denote the angle between the normal plane vector $\vec{n}$ and horizontal plane and *ρ*, respectively. The upright axis denotes the convergence degree of each candidate parameter set. The planar parameters statistical test compares the angle between hypothesis planar normal vectors to the deviation between hypothesis *ρ* s.

**Fig 6:**
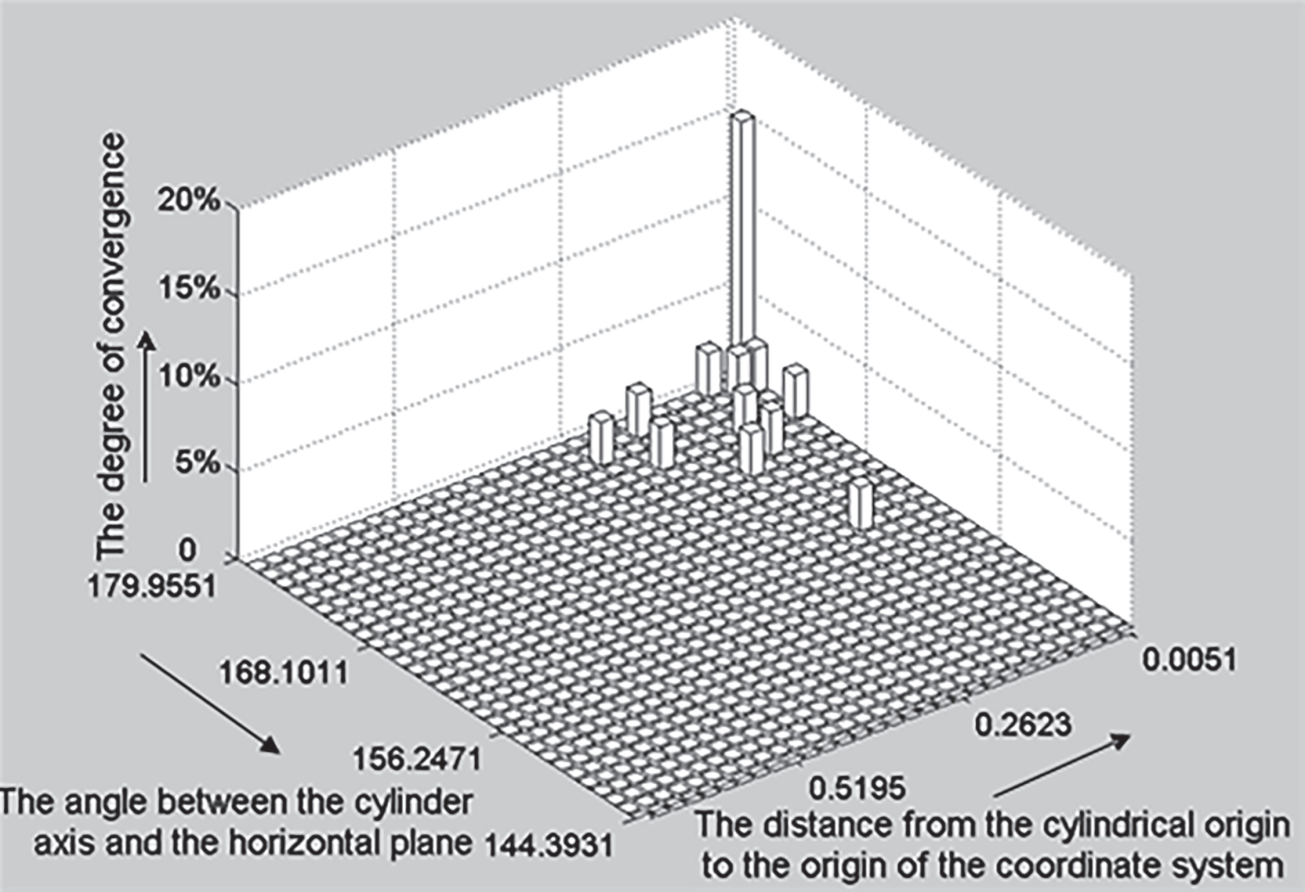
The 2D histogram of a cylinder. The horizontal and vertical axes denote the angle between the cylinder axis and horizontal plane and the distance from the cylindrical origin to the origin of the coordinate system respectively; the upright axis represents the convergence degree of each candidate parameter set. This degree of candidate parameter set convergence in the histogram is calculated as the number of parameter sets that converge to the candidate parameter set divided by the total number of parameter sets.

**b) The 2D histogram of a cylinder.** To determine the degree of deviation between hypothesis parameter sets, we constructed a vector with seven dimensions for each set of cylindrical parameters and computed the Euclidian distances between different vectors. We used a 2D histogram (Fig. 6) to visualize the statistical testing process. Consequently, the seven parameters were reduced to two, i.e., the angle between the cylinder axis and horizontal plane (horizontal axis) and the distance from the cylindrical origin to the origin of the coordinate system (vertical axis). The upright axis represents the convergence degree of each candidate parameter set.

**Fig 7:**
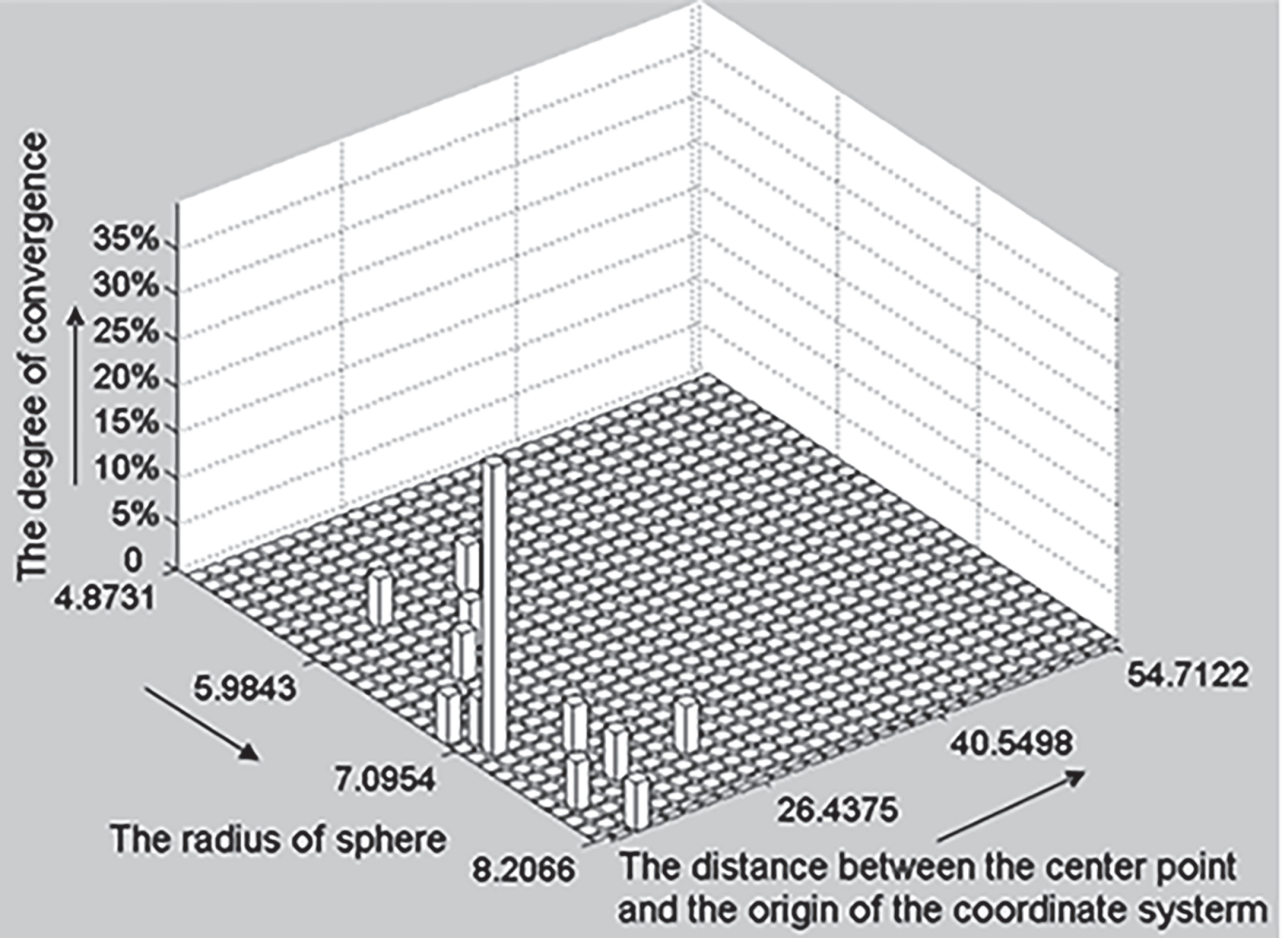
The 2D histogram of a sphere. The horizontal and vertical axes denote the distance between the hypothesis center point and coordinate system origin and the hypothesis radius respectively; the upright axis represents the convergence degree of each candidate parameter set. This degree of candidate parameter set convergence in the histogram is calculated as the number of parameter sets that converge to the candidate parameter set divided by the total number of parameter sets.

**c) The 2D histogram of a sphere.** According to Section A, the differences between spherical hypothesis parameter sets were evaluated in light of hypothesis center points and radii. In Fig. 7, the horizontal and vertical axes represent the distance between the hypothesis center point and coordinate system origin and hypothesis radius, respectively. The upright axis represents the convergence degree of each set of hypothesis spherical parameters.

**d) Initial multiple primitive detection.** The hypothesis testing process begins with an RANSAC strategy through which initial datasets are randomly selected. During each iteration, the histogram of the candidate parameter sets was updated using the newly calculated hypothesis parameter set. If the new set does not converge to any existing candidate parameter set, it is considered a new candidate in the histogram. Otherwise, we increased the degree of convergence of the existing candidate set. If the tested point cloud contains multiple primitives, the peaks of the convergence degree (e.g., in Fig. 7) can be found after a certain number of iterations. Each peak that reaches a predefined threshold denotes a potential primitive model. In this way, initial multiple primitives can be detected before verifying the entire point cloud using the hypothesis primitive model.

### E) Fitting of multiple primitives based using the multiBaySAC algorithm

After the initial multiple primitives were identified, we conducted multiple model fittings using BaySAC and ran the following conditional sampling process for each initial primitive during each iteration.

The hypothesis set in the BaySAC algorithm that is the most likely to be correct was selected, and this was determined based on prior inlier probabilities of data points.

**a) Prior inlier probability estimation.** Initial primitive parameters were employed to determine prior data point probabilities. Equation (7) describes the strategy for computing the prior probabilities:

$$P_{i}=\{\begin{array}{cc} 1-\frac{D_{i}}{m} & \left(D_{i}<m\right)\\ 0 & \left(D_{i}>=m\right) \end{array}\;,$$ (7)

where *P*_*i*_ denotes the prior probability of point i, *D*_*i*_ is the distance between point i and the fitted primitive, and m represents the predefined threshold for outlier identification, which is set as five times the value of the point precision.

In the multiBaySAC algorithm, a data point was assigned to multiple prior probabilities for the initial multiple primitives. Each denotes the probability that a point belongs to its corresponding primitive.

**b) Hypothesis model verification.** The hypothesis testing process was implemented using the BaySAC strategy. For each potential primitive, we selected the hypothesis sets with highest prior probabilities (e.g., four points for a plane) to compute the hypothesis primitive parameters. As noted in Section C(a), multiple prior probabilities were assigned to each data point, and each denotes the probability that a point belongs to the potential primitive. Therefore, we selected the data point with the highest prior probability regarding the current hypothesis primitive as determined in terms of the current hypothesis primitive and based on multiple prior probabilities assigned to the considered point. Hypothesis primitive parameters were then computed using the selected hypothesis sets. We verified each data point by its point-to-primitive distance to determine its compatibility with the hypothesis model (if its point-to-primitive distance is smaller than the predefined threshold, the answer is yes). However, it is likely that the considered point will pass the verification over more than one hypothesis primitive due to the possible intersection between different primitives. In such cases, the point was assigned to the primitive from which the highest prior probability is calculated.

During the BaySAC hypothesis testing process, inlier probabilities of the current hypothesis set were updated with each iteration based on current hypothesis model verification results..

**c) Probability updating.** The probability updating principle of the BAYSAC algorithm [21] is as follows:

$$P_{t}\left(i\in I\right)=\{\begin{array}{c} \frac{P_{t-1}\left(i\in I\right)P\left(H_{t}⊄I|i\in I\right)}{P\left(H_{t}⊄I\right)},i\in H_{t}\\ P_{t-1}\left(i\in I\right),i\notin H_{t} \end{array}$$ (8)

where *I* is the set of all inliers; *H*_*t*_ is the hypothesis set of n data points used for iteration t of the hypothesis testing process; *P*_*t−1*_ *(i*ε*I)* and *P*_*t*_ *(i*ε*I)* denote the inlier probability for data point i at iteration t−1 and t, respectively; *P(H*_*t*_ *⊄I)* denotes the probability of the presence of outliers in the hypothesis set; and *P(H*_*t*_ *⊄I | i*ε*I)* denotes the probability of the presence of outliers in the hypothesis set under the condition that point i is an inlier.

Equation (9) represents a form of Bayes’ Theorem, which states that the posterior *P*_*t*_ *(i*ε*I)* is proportional to the prior *P*_*t−1*_ *(i*ε*I)* times the likelihood. For further details on the deduction of Equation (9), please refer to [23].

$$P_{t}\left(i\in I\right)\propto P_{t-1}\left(i\in I\right)\times \mathrm{Likelihood}$$ (9)

Here, the term *likelihood* implies that the posterior is a function of the prior. The symbol ∝ denotes the proportional relationship between the two events.

Because the probability that the hypothesis model is the best model can be used to evaluate the probability that the corresponding hypothesis set is correct, we describe the likelihood using Equation (10):

Likelihood≈k/D, (10)

where k is the number of points consistent with the model during a test and D is the total number of data points. In turn, we obtained the following simplified formula for probability updating:

$$P_{t}\left(i\in I\right)=\{\begin{array}{cc} \frac{k}{D}P_{t-1}\left(i\in I\right) & i\in H_{t}\\ P_{t-1}\left(i\in I\right) & i\notin H_{t} \end{array}.$$ (11)

**Fig 8:**
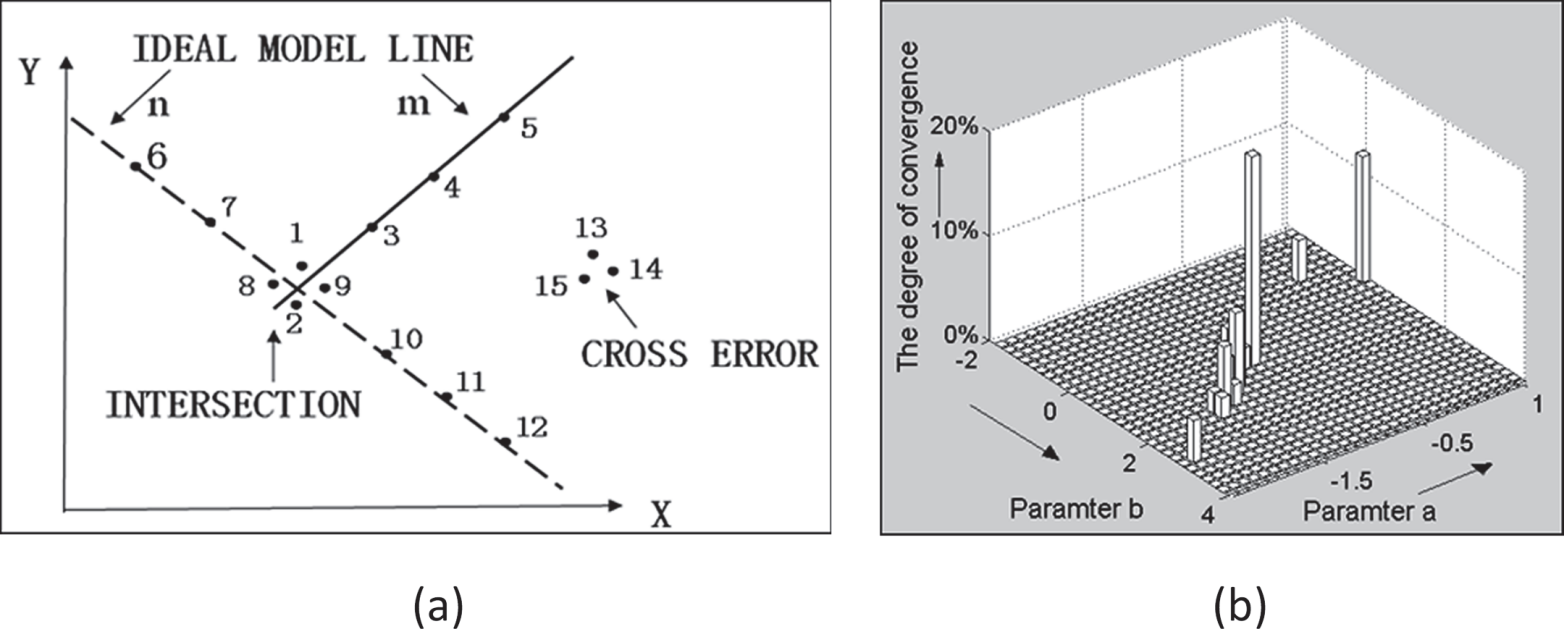
An example of the proposed multiBaySAC. (**a**) Fifteen candidate points for fitting two lines that contain three outliers, i.e. points 13, 14 and 15 (**b**) The detection of potential lines using the hypothesis model parameters histogram.

**d) Algorithm process.** We illustrated the multiBaySAC algorithm process using a brief example involving the fit between two lines. Fifteen candidate points for fitting two lines are shown in Fig. 8. There outliers were included, i.e., points 13, 14 and 15.

The process began with the proposed statistical testing strategy for detecting potential lines. An initial dataset of two points was randomly selected from the 15 candidate points using an RANSAC strategy. Meanwhile, the proposed statistical testing of the candidate line parameter set was iteratively implemented using the newly calculated hypothesis parameter set. We computed the degree of convergence of a candidate parameter set based on the angle between the direction vectors of lines fitted during different iterations. After 35 iterations, the two highest degrees of convergence were found, exceeding the predefined threshold of 15%; thus, their corresponding line parameters were used to determine the prior probabilities of the 15 points using Equation (7) (Table 1). As noted in Section A, each data point was assigned to two prior probabilities for the two detected initial lines. Each describes the probability that a point belongs to its corresponding line. We can see that the computed prior probability of the three outliers is zero.

**Table 1:** An example of probability updating.

Point (D = 15)	Prior inlier probabilities		Probabilities after the first update		Probabilities after the second update	
	Line m	Line n	Line m (K = 6)	Line n (K = 6)	Line m (K = 7)	Line n (K = 9)
1	**0.9999**	0.9512	0.2001	**0.9512**	0.2001	0.3675
2	0.7879	0.3571	**0.7879**	0.3571	0.0083	0.3571
3	0.3577	0.0000	0.3577	0.0000	0.3577	0.0000
4	**0.9999**	0.0000	0.2001	0.0000	**0.4999**	0.0000
5	0.9953	0.0000	**0.9953**	0.0000	**0.4561**	0.0000
6	0.0000	**0.9999**	0.0000	0.2001	0.0000	0.3999
7	0.0000	0.9189	0.0000	**0.9189**	0.0000	0.2136
8	0.7949	0.8308	0.7949	0.8308	0.7949	0.8308
9	0.6747	0.8101	0.6747	0.8101	0.6747	0.8101
10	0.0000	0.8873	0.0000	0.8873	0.0000	**0.8873**
11	0.0000	**0.9999**	0.0000	0.2001	0.0000	0.3999
12	0.0000	0.8873	0.0000	0.8873	0.0000	**0.8873**
13	0.0000	0.0000	0.0000	0.0000	0.0000	0.0000
14	0.0000	0.0000	0.0000	0.0000	0.0000	0.0000
15	0.0000	0.0000	0.0000	0.0000	0.0000	0.0000

Hypothesis testing processes were then implemented in conjunction with the BaySAC strategy for the two detected initial lines. When fitting line m, we selected points 1 and 4, which have the two highest probabilities, to calculate line parameters with which all points were then tested. There were six inlier points. The probabilities of points 1 and 4 were then updated based on Equation (11), completing the first iteration of BaySAC for line m. During the second iteration, the points with the two highest probabilities should be points 5 and 8; however, the probability of point 8 for line m is less than that of line n. As a result, point 8 was replaced with point 2, whose probability for line m is the third highest. Hypothesis line parameters were computed using points 2 and 5, and seven inlier points were selected, implying that we had identified the best model that is consistent with all inliers. The probabilities of points 2 and 5 were also updated based on Equation (11). In the third iteration, points 4 and 5 were selected, and the hypothesis process repeated. However, because the best model found during the second iteration is consistent with all inliers, no hypothesis line model that was identified during successive iterations can exceed it. As a result, the hypothesis testing process is complete when the number of sampling iterations reaches the predefined threshold.

Of the inliers, points 1, 2, 8 and 9 were assigned to both fitted lines because they were positioned close to the point of intersection between the two lines. As noted in Section C(b), such points were reassigned to the line with the highest inlier probability. In Table 1, the probabilities of line n assigned to the four points are all higher than those of line m after the second updating stage. However, for point 2, the prior inlier probability of line m, 0.7879, is larger than that of line n, 0.3571. Moreover, point 2 was not selected for model hypothesis testing for line n. We consequently set point 2 to line m and assigned the other points to line n.

## Experimental Results

**Fig 9:**
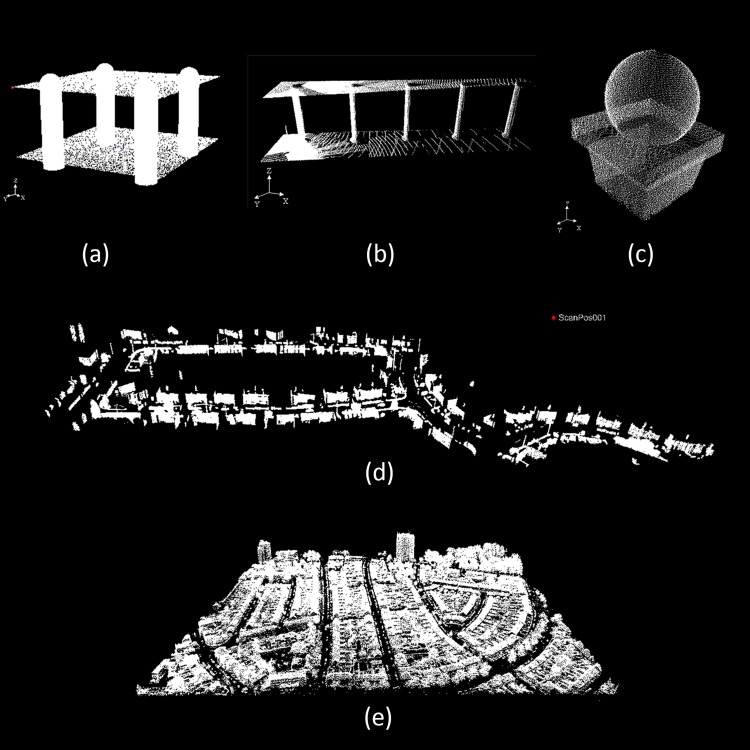
Test point clouds. (**a**) Synthetic Dataset I includes two planes, four spheres and four cylinders;(**b**) Real Dataset II includes two planes and five cylinders **(c)** Real Dataset III includes several planar primitives and one spherical primitive **(d)** Real Dataset IV includes several planar primitives **(e)**Real Dataset V consists of an airborne point cloud with planar roofs.

As a traditional method that addresses multiple models, the sequential RANSAC framework involves consecutively applying the standard RANSAC model and then removing detected inliers. Therefore, to evaluate the proposed algorithm, the performance of the multiBaySAC algorithm was compared to that of the sequential RANSAC using real and synthetic datasets (Fig. 9) with respect to computational efficiency and fitting accuracy. The synthetic data (Dataset I) include two planes, four spheres and four cylinders. The real datasets (Datasets II-V) include point clouds that capture scenes of buildings with cylindrical primitives, spherical primitives and numerous planar primitives acquired via terrestrial, airborne and vehicle-based LiDAR, respectively. Datasets II and III were acquired using a RIEGL LMS-Z620 laser scanner at a university campus facility. Dataset IV, which covered a residential area, was acquired using a LYNX mobile mapping system. Dataset V was obtained using the Fugro FLI-MAP airborne laser scanning system in an urban environment. Table 2 describes the experimental data.

**Table 2:** Description of test point clouds.

Point cloud	Number of points	Primitive captured
Dataset I, synthetic	476354	Planes, spheres, cylinders
Dataset II, Terrestrial LiDAR	30307	Planes, spheres
Dataset III, Terrestrial LiDAR	146030	Planes, cylinders
Dataset IV, Vehicle-based LiDAR	1979896	Planes
Dataset V, Airborne LiDAR	450298	Planes

### A) Initial multiple primitive detection

**Fig 10:**
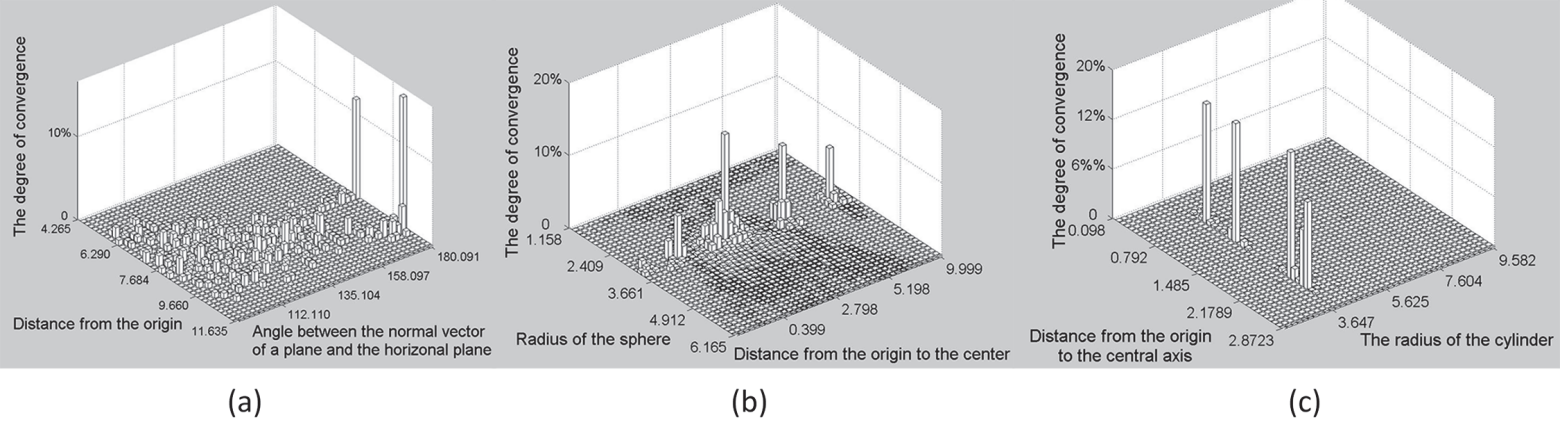
Potential multiple primitive detection (Dataset I). (**a**) Two detected peaks of planar parameter sets (**b**) Four detected peaks of spherical parameter sets **(c)** Four detected peaks of cylindrical parameter sets.

As noted in Section II B, candidate model parameters statistical testing was employed to detect initial multiple primitives. For instance, Fig. 9A shows that Dataset I includes two planes, four spheres and four cylinders. An iterative process that randomly selected initial datasets was initiated. During each iteration, we selected three initial datasets simultaneously to compute new hypothesis parameter sets for a plane, sphere and cylinder, with which the three histograms (Fig. 10) of the candidate parameter sets were updated as shown in Section II C(d). The threshold of the convergence degree was set as 8%, denoting that if a peak of the convergence degree peak reaches 8%, its corresponding candidate parameter set is detected as a potential primitive model. As illustrated in Fig. 10, two peaks in (a), four peaks in (b) and four peaks in (c) were identified in agreement with the number and types of primitives included in Dataset I.

**Fig 11:**
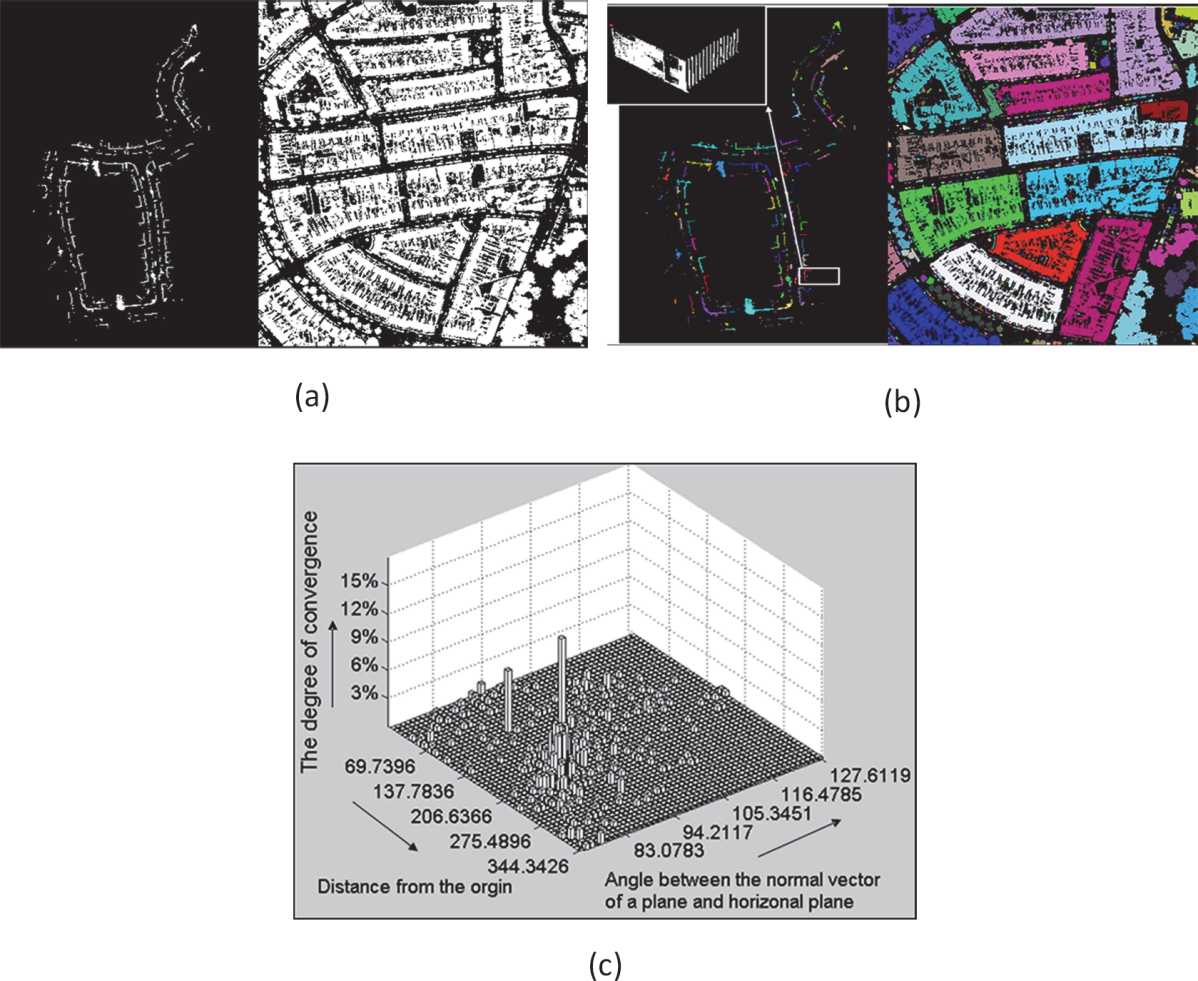
Potential multiple primitive detection (Dataset IV and V) (**a**) 2D point-density images: Dataset IV (Left) and Dataset V (Right); (b) Segmentation results: Dataset IV (Left) and Dataset V (Right); (c) Statistical testing of candidate model parameters for the highlighted segment of Dataset IV.

Because Datasets IV and V contain numerous primitives, it is very difficult and time-consuming to directly detect initial multiple primitives via candidate model parameters statistical testing. Therefore, we pre-segmented the two datasets and then detected respective initial primitives for each segment. Prior to pre-segmentation, ground points were filtered out through their corresponding z coordinate as ground points have relatively small z values, and their neighbors have a similarly low z values. The pre-segmentation process involves two steps: 2D point-density image generation and image segmentation via connectivity analysis. As shown in Fig. 11A, the point cloud was projected onto the XOY plane and the divided into cells of 10 x 10 cm.A gray-scale image in which gray values were scaled in terms of the number of laser points within a cell was then generated. The region-growing algorithm was employed to segment the 2D point-density image using pixel-to-pixel connectivity as the growing criterion. Fig. 11B shows the segmentation results for Datasets IV and V, in which different colors represent different segments. For each segment, candidate model parameters statistical testing was applied to detect initial multiple primitives (for instance, two peaks were identified from the segment highlighted in the white box in Fig. 11B (Left)).

### B) Fitting accuracy

**Fig 12:**
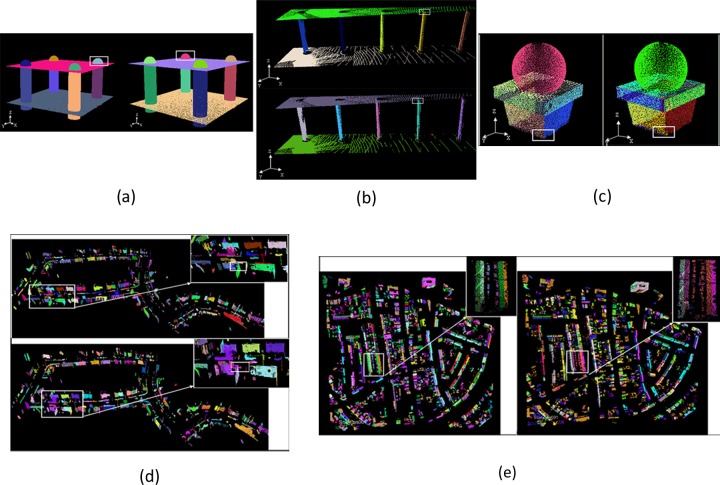
Fitting results. The white box highlights the intersection between two primitives; the left/upper sections show multiBaySAC results and the right/lower sections illustrate sequential RANSAC results: **(a)** Dataset I; **(b)** Dataset II; **(c)** Dataset III; **(d)** Dataset IV; **(e)** Dataset V.

Fig. 12 shows the fitting results of the proposed multiBaySAC algorithm and the sequential RANSAC framework. Different colors represent different inlier sets that comply with the fitted primitives.

**Fig 13:**
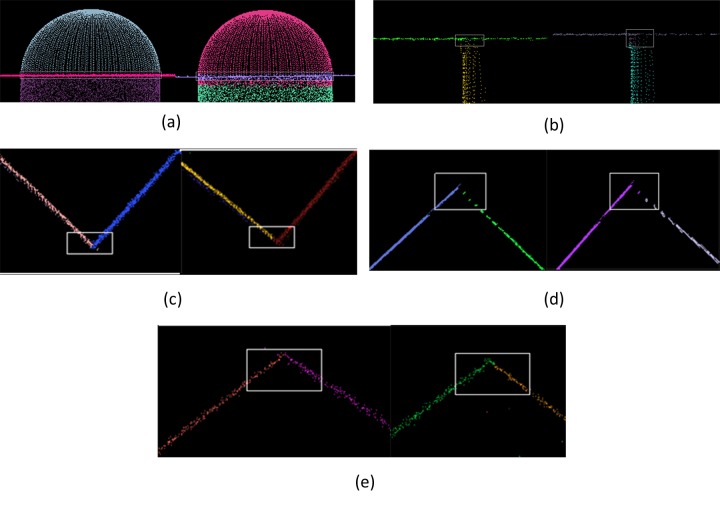
Fitting performances at the intersection between two primitives. The left-hand section shows multiBaySAC results and the right-hand section illustrates sequential RANSAC results: **(a)** Dataset I; **(b)** Dataset II; **(c)** Dataset III; **(d)** Dataset IV; **(e)** Dataset V.

As noted previously, we consecutively applied standard RANSAC to the experimental datasets. However, because the previously detected set of inliers was removed from the raw data, the intersection of two primitives (highlighted in the white box in Fig. 12) presents both over- and under-segmentation problems (Fig. 13 (right-hand section)). In Fig. 13 (right-hand section), the inlier set of the previously fitted primitive includes points that should belong to the primitive that was fitted subsequently. This occurred because the threshold for accepting a point as an inlier can include other points positioned close to the intersection between two primitives. To solve this problem, we applied BaySAC procedures to the detected initial primitive models. Before initiating the BaySAC process, multiple prior inlier probabilities were assigned to each data point, which were computed using Equation (7) in terms of the detected initial primitive models. We then updated the prior inlier probabilities throughout the multiBaySAC process. In turn, the point considered was assigned to the primitive with which the highest inlier probability was found when the point passed over more than one verification of the hypothesis primitive. As shown in Fig. 13 (left-hand section), points positioned close to the intersection between two primitives were more correctly assigned relative to the results acquired via the sequential RANSAC procedure (Fig. 13 (right-hand section)).

Table 3 lists the numeric fitting results of the sequential RANSAC and multiBaySAC for Datasets IV and V (Fig. 12 (D) and (E)). The fitting accuracies were evaluated via the IOR (Inlier-to-Outlier Ratio).

**Table 3:** Numeric fitting results of Datasets IV and V.

Dataset	Algorithm	Fitted Primitives	Correct Primitives	Wrong Primitives	IOR
IV	Sequential RANSAC	734	695	39	17.82
	MultiBaySAC	759	715	44	16.25
V	Sequential RANSAC	901	856	45	19.02
	MultiBaySAC	1015	973	42	23.17

As shown in Table 3, MultiBaySAC demonstrates its robustness across all of the experiments conducted because it fitted more correct primitives than Sequential RANSAC.

### C) Computational efficiency

Hypothesis testing is an iterative process; therefore, the computational efficiencies of the proposed strategies were evaluated in terms of the number of iterations performed. As described in Section II, the iterations of multiBaySAC include two components, i.e.,iterations performed for the detection of initial multiple primitives (Part 1 of S1 Table) and iterations performed for parallel BaySAC processes (Part 2 of S1 Table). It is shown in S1 Table that Part 1 of multiBaySAC dominates the computational cost of the process. Once the initial primitive models are detected and prior inlier probabilities of data points are computed, the number of iterations used to calculate the final model (Part 2) is significantly decreased. S1 Table also shows that our algorithm improved average computational efficiency values 34% more than the sequential RANSAC method.

## Discussion

RANSAC is a well-regarded technique for the segmentation and robust model fitting of range data, because it is proven to be capable of managing more than 50% of all outliers. The computational cost of RANSAC is proportional to the number of iterations that are implemented before a sufficient model is found. However, the original RANSAC framework assumes that all data points are equally probable, and thus many hypothesis sets will be contaminated with outliers when the percentage of outliers is high, generating numerous iterations and high computational costs. The proposed multiBaySAC algorithm implemented a conditional sampling strategy that introduces inlier probabilities to the hypothesis testing process. Inlier probabilities describe how a data point relates to its corresponding primitive. As our strategy always selects the minimum number of data points required with the highest inlier probabilities as a hypothesis set and thereby reduces the number of iterations needed to determine a good model computational costs can be significantly reduced.

Moreover, the original RANSAC method assumes that a single model accounts for all data inliers, precluding it from processing data containing more than one model. To address this issue, the sequential strategy (sequential RANSAC) has been often used to manage multiple models (or different instances of the same model). However, over- or under-segmentation problems may occur when standard RANSAC is consecutively implemented. Our proposed multiBaySAC performed hypothesis testing processes simultaneously for the detected initial primitive models. During this process, multiple inlier probabilities were assigned to each data point. In turn, the considered point was assigned to the primitive with the highest inlier probability when the point passes over more than one verification of the hypothesis primitive. This strategy can thus prevent problems of inlier misidentification that are common of sequential methods.

The multiBaySAC algorithm presented here is optimized from the BaySAC algorithm (Bayes Sample Consensus) [21]. It is important that the BaySAC algorithm effectively determines prior inlier probability values. In the original BaySAC paper [21], prior probabilities were either constant (e.g. 0.5) or were randomly drawn from a uniform distribution (e.g., 0.25, 0.75). However, the original paper disclosed the possibility that degenerate configurations that are incorrectly assumed to contain outliers could cause a sampling strategy to fail. Moreover, the original BaySAC method is only proposed to estimate a single model, the fundamental matrix, from an image pair. To improve upon the robustness and applicability of the original BaySAC method, we extend this method to the multiBaySAC method. A novel approach to the statistical testing of candidate model parameters was first presented to detect initial multiple primitives, with which multiple prior inlier probabilities were computed and assigned to each data point. The detected primitives were then optimized using a parallel rather than sequential strategy. Moreover, the prior probability was updated using the simplified Bayes’ formula, and the probability that describes the probability that the corresponding hypothesis set is fully correct.

## Conclusions

In this paper, we present the multiBaySAC algorithm as a means of fitting multiple primitives from unorganized 3D point clouds. First, a novel algorithm for the statistical testing of candidate model parameters is presented to detect potential multiple primitives before performing a thorough hypothesis testing procedure on the point cloud. Multiple prior inlier probabilities for each data point are determined using point-to-primitive distances that were computed in terms of detected initial multiple primitives. We employ a parallel rather than sequential strategy to implement the BaySAC process for optimizing initial primitive models. BaySAC inlier probability updating is simplified based on a frequently used form of Bayes’ Theorem that compares prior and posterior probabilities of a data point by considering the probability that the hypothesis set of a data point is correct.

The proposed algorithms are implemented and evaluated based on their computational efficiency and based on the quality of fitting results using both real and synthetic datasets. The results show that multiBaySAC achieved better results than sequential RANSAC with respect to the intersection between two primitives, for which the sequential RANSAC framework is expected to suffer from both over- and under-segmentation problems. Moreover, the results illustrate that our proposed algorithm improved computational efficiency levels by 34% on average relative to the performance of the sequential RANSAC framework. This optimization is attributable to the parallel strategy that was used for hypothesis testing and to the BaySAC algorithm, which effectively prevented the development of useless trials that characterize the RANSAC random selection strategy of RANSAC.

The proposed multiBaySAC approach, which is based on the statistical testing of candidate model parameters, is independent of the particular geometric setting, thus making it applicable to any problem that currently utilizes RANSAC. Therefore, future work must further optimize this strategy by applying it to other problems such as multiple point cloud co-registration and multiple image matching.

## Supporting Information

S1 Table The comparison of computational efficiencies. (PDF)
