# Supplementary material for: Primitive Fitting Based on the Efficient multiBaySAC Algorithm
Source: PLoS One. 2015 Mar 17;10(3):e0117341. doi: 10.1371/journal.pone.0117341 (PMC4363901; doi:10.1371/journal.pone.0117341)
Supplement: S1 Table — (PDF) [file pone.0117341.s001.pdf]

Table S1. The comparison of computational efficiencies

Dataset	Number of points	Primitive	Sequential RANSAC		MultiBaySAC		
			Iterations		Iterations		
			Total	each plane	Total	Part 1	Part 2
I	476354	P_{I-1}	37830	329	18548	369	3
		P_{I-2}		295			2
		S_{I-1}		10685		17253	3
		S_{I-2}		9662			4
		S_{I-3}		8323			2
		S_{I-4}		6587			4
		C_{I-1}		628		995	2
		C_{I-2}		595			6
		C_{I-3}		421			2
		C_{I-4}		305			3
II	146030	P_{II-1}	591	255	478	465	5
		P_{II-2}		336			8
		C_{II-1}	10091	528	7791	7760	8
		C_{II-2}		645			4
		C_{II-3}		1782			6
		C_{II-4}		4943			6
		C_{II-5}		2193			7
III	30307	C_{III-1}	821	821	476	471	5
		P_{III-1}	4896	1548	3471	3449	8

		P_{III-2}		1261			2
		P_{III-3}		1382			7
		P_{III-4}		705			5
VI	1979896	P_{VI-1}-$P_{VI-734/759}$	12534966	\	8713513	8700784	12729
V	450298	P_{V-1}-$P_{V-901/1015}$	21577809	\	13987579	13943194	44385

Note: P-Plane; C-Cylinder; S-Sphere
